# From Culturomics to Clinical Microbiology and Forward

**DOI:** 10.3201/eid2409.170995

**Published:** 2018-09

**Authors:** Grégory Dubourg, Sophie Baron, Frédéric Cadoret, Carine Couderc, Pierre-Edouard Fournier, Jean-Christophe Lagier, Didier Raoult

**Affiliations:** Aix Marseille University, Institut de Recherche pour le Développement (IRD), Assistance Publique des Hôpitaux de Marseille (APHM), Microbes, Evolution, Phylogeny and Infections (MEPHI), Institut Hospitalo-Universitaire (IHU) Méditerranée Infection, Marseille, France (G. Dubourg, S. Baron, J.-C. Lagier, D. Raoult);; APHM, IHU Méditerranée Infection, Marseille (F. Cadoret, C. Couderc);; Aix Marseille University, IRD, APHM, Vecteurs–Infections Tropicales et Méditerranéennes, IHU Méditerranée Infection, Marseille (P.-E. Fournier)

**Keywords:** culturomics, MALDI-TOF MS, new species, clinical microbiology, culture, microbiology, bacteria, matrix-assisted laser desorption/ionization time-of-flight mass spectrometry

## Abstract

Culturomics has permitted discovery of hundreds of new bacterial species isolated from the human microbiome. Profiles generated by using matrix-assisted laser desorption/ionization time-of-flight (MALDI-TOF) mass spectrometry have been added to the mass spectrometer database used in clinical microbiology laboratories. We retrospectively collected raw data from MALDI-TOF mass spectrometry used routinely in our laboratory in Marseille, France, during January 2012–March 2018 and analyzed 16S rDNA sequencing results from misidentified strains. During the study period, 744 species were identified from clinical specimens, of which 21 were species first isolated from culturomics. This collection involved 105 clinical specimens, accounting for 98 patients. In 64 cases, isolation of the bacteria was considered clinically relevant. MALDI-TOF mass spectrometry was able to identify the species in 95.2% of the 105 specimens. While contributing to the extension of the bacterial repertoire associated with humans, culturomics studies also enlarge the spectrum of prokaryotes involved in infectious diseases.

The diagnosis of bacterial diseases in clinical microbiology has relied on phenotypic identification, based on the bacterial repertoire known to be associated with humans. This mode of identification, which is, in fact, recognition of previously described microorganisms, does not allow for the identification of new bacteria. Recently, the systematic use of universal 16S rDNA gene sequencing of cultivated bacteria that presented an atypical phenotypical profile paved the way for identifying rare, fastidious, and new microorganisms (*1**,**2*)*.* However, this method implies redefining specific phenotypical characteristics, which sometimes cannot be done because of the limited number of available biochemical tests. More recently, the revolution provided by matrix-assisted laser desorption/ionization time-of-flight (MALDI-TOF) mass spectrometry identification permits comparison of a protein spectrum obtained from a colony with a database, which can be permanently incremented with newly identified bacteria (*3**,**4*)*.* The use of a cutoff identification score, with values in the range of 1.7–2, enables correct identification of the isolate. However, when MALDI-TOF mass spectrometry recognizes bacteria never previously associated with humans, it is reasonable to carry out confirmation by sequencing the 16S rDNA gene. The main advantage of MALDI-TOF mass spectrometry compared with sequencing methods is that it is extremely fast and cost-effective (*3**,**4*)*.* Indeed, the cost involves mainly the cost of the machine; the individual cost per test is insignificant. Thus, the ease in testing bacterial colonies led us to establish the repertoire of commensal bacteria of the human microbiota in the laboratory at IHU Méditerranée Infection in Marseille, France, by using a high-throughput culture and MALDI-TOF mass spectrometry identification. Sequencing of the 16S rDNA gene enables identification of atypical bacteria with definition of new bacterial species, whose genomes are then sequenced. This approach, called culturomics (*5**,**6*), has made possible the addition of 672 bacteria to the known repertoire of the bacteria already isolated from the human mucosa. Other teams, in parallel, have used similar approaches (*7**,**8*)*.*

The usefulness of culturomics in increasing knowledge of the repertoire of cultivable bacteria from human mucous membranes appears clear for microbiota studies. However, the benefit of this process in clinical microbiology is prone to controversy. We speculated that commensal bacteria found in humans may be involved in opportunistic infections. In our experience, the creation of new spectra enabled us to increment our MALDI-TOF mass spectrometry database used for clinical microbiology, thus enabling recognition of bacterial species first isolated as a part of culturomics studies and improving the accuracy of diagnosis of infectious diseases involving bacteria.

## Materials and Methods

### Settings

All data included in this study were obtained from the routine microbiology laboratory at IHU Méditerranée Infection, which receives a mean annual number of 350,400 samples from the 4 Marseille university hospitals (Timone, Conception, North, and Sainte-Marguerite hospitals), which contain a total of 3,700 beds. Retrospective data were collected for January 2012–March 2018.

### Routine Bacteriological Practices

We analyzed samples according to standard microbiological procedures, as previously described, depending on the specimen (*9**–**12*)*.* This process included systematic inoculation onto Columbia agar with 5% sheep blood (BioMérieux, Craponne, France), chocolate agar (BioMérieux) (excluding urine and fecal samples), and specific media such as colistin-nalidixic agar or MacConkey agar (both BioMérieux) for specimens potentially contaminated by resident flora. Blood cultures were incubated into a Bactec device (Becton Dickinson, Le Pont de Claix, France) and analyzed as previously described (*9*).

### Specific Cultures

We plated fecal specimens taken following a regional outbreak of *Clostridioides* (formerly *Clostridium*) *difficile* 027 during May 2013–March 2018 (*13*), in which toxin detection was positive using GeneXpert *C. difficile* PCR (Cepheid, Paris, France) after ethanol treatment (*14*), to obtain *C. difficile* isolates. We also investigated possible multidrug-resistant bacteria carriage by plating on chromID MRSA agar for methicillin-resistant *Staphylococcus aureus*, chromID CARBA SMART medium (BioMérieux) for carbapenemase-producing *Enterobacteriaceae* (CPE), and Drigalski/MacConkey agar (BioMérieux) for third-generation, cephalosporin-resistant, gram-negative bacteria.

### Identification of Colonies

We performed bacterial identification on colonies using MALDI-TOF mass spectrometry, as previously described (*3**,**4*)*.* We considered identification to be correct when the identification score was >1.9 and when the same single species was recognized. When identification did not meet these criteria, we performed proteic extraction using formic acid and acetonitrile (*15*)*.* If identification was still incorrect following the proteic extraction protocol, we performed 16S rDNA sequencing systematically, as previously described (*16*), in 3 situations: when the identification score was <1.9 despite proteic extraction, when multiple different species were recognized with a correct identification score, and when a bacterium was isolated for the first time in the clinical microbiology laboratory.

### Culturomics Studies

In brief, culturomics consists of the multiplication of culture conditions applied to human specimens to increase the repertoire of the human microbiome. The pioneering study used 212 conditions (*5*); this number was reduced to 70 in 2012 (*17**,**18*) and then to 18 in 2014 (*6*)*.* In addition, several specific conditions were designed for archaea, microcolonies, proteobacteria, and microaerophilic and halophilic bacteria. Most specimens used were fecal samples. However, respiratory, vaginal, and urine samples have been analyzed recently in the context of culturomics studies. Identification has also been performed using MALDI-TOF mass spectrometry. Colonies were considered correctly identified when 2 colonies exhibited an identification score >1.9. If identification scores were not correct after 3 attempts, sequencing of the 16S rDNA gene was performed (*16*)*.* If there was <98.7% similarity with the closest neighbor, the bacterial isolate was considered to be a new species (*19*).

### Updating the MALDI-TOF Mass Spectrometry Database

The database used for routine bacterial identification is updated through 3 sources: updates from the MALDI-TOF mass spectrometry manufacturer, updates from culturomics studies, and routine laboratory results. Updates from culturomics studies and routine laboratory results are based on 16S rDNA sequencing results.

### Analysis of Data from MALDI-TOF Mass Spectrometry Used in the Clinical Microbiology Laboratory

We retrospectively collected raw data from MALDI-TOF mass spectrometry used in the microbiology laboratory involving identifications performed during January 2012–March 2018, which are saved monthly. Data were deduced from the samples. These data do not consider the clinical relevance of the identified microorganism, the final result, or multiple attempts to identify the colony using MALDI-TOF mass spectrometry.

## Results

### Bacterial Identification in Clinical Microbiology Laboratory

During January 2012–March 2018, the clinical microbiology laboratory performed 351,937 nondereplicated bacterial identifications using MALDI-TOF mass spectrometry. Of these, 28,391 (8.1%) were unidentified or misidentified. When we looked at the yearly ratio of unidentified bacteria, we noticed that it fell from 17.7% in 2012 to 3.6% in 2018 (Figure). Overall, we identified 744 unique bacterial species correctly using MALDI-TOF mass spectrometry.

**Figure:**
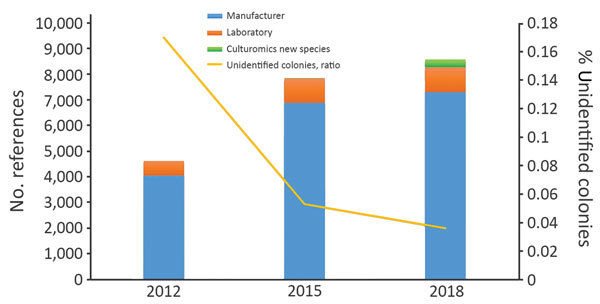
Annual ratio of unidentified bacteria and evolution of the number of spectral references available in the matrix-assisted laser desorption/ionization time-of-flight mass spectrometry database in a clinical laboratory in Marseille, France.

### Contribution to MALDI-TOF Mass Spectrometry Database Updates

During the study period, we added 4,539 references to our database. Updates from the manufacturer comprised 3,255 references, whereas 983 references came from routine laboratory results. In addition, 306 (23.4%) updates were from new bacterial species discovered as a part of culturomics studies. Overall, references from the manufacturer represented 87% of the total database, routine laboratory results represented 8%, and new culturomics species represented 5% (Figure).

### Routine Identification of Species Isolated as Part of Culturomics Studies

Among the 351,937 bacterial identifications performed routinely during the study period, we identified species first isolated from culturomics studies in 105 clinical specimens, accounting for 98 patients. This collection represents a total of 21 species, accounting for 2.8% (21/744) of the overall microbiology laboratory bacterial diversity (Table 1).

**Table 1:** Main features of the bacteria discovered as part of culturomics studies identified in a clinical microbiology laboratory*

Species	Culturomics study	CSUR no.	Strain	GenBank accession no.	Date of spectrum implementation	No. cases	References
*Actinomyces bouchesdurhonensis*	Gut microbiota (storied samples)	P2825	Marseille-P2825T	LT576385	2017 Apr	3	Unpub. data
*Actinomyces ihuae*	Gut microbiota (HIV)	P2006	SD1	LN866997	2015 Jul	17	(*6**,**20*)
*Actinomyces marseillensis*	Respiratory microbiota	P2818	Marseille-P2818T	LT576400	Not added	1	(*21*)
*Alistipes jeddahensis*	Gut microbiota	P1209	AL1	LK021116	2015 Oct	4	(*6**,**22*)
*Anaerosalibacter massiliensis*	Gut microbiota (Polynesia)	P762	ND1	HG315673	2013 Apr	1	(*6**,**23*)
*Bacteroides timonensis*	Gut microbiota (anorexia nervosa)	P194	AP1	JX041639	2016 Apr	2	(*6**,**24*)
*Butyricimonas phocaeensis*	Gut microbiota (obese)	P2478	AT9	LN881597	2015 Nov	1	(*6**,**25*)
*Clostridium culturomicsense*	Gut microbiota (Saudian obese)	P1184	CL6	LK021117	2014 Sep	1	(*6**,**26*)
*Clostridium jeddahtimonense*	Gut microbiota (obese)	P1230	CL2	LK021118	2014 Aug	7	(*6*)
*Clostridium massilioamazoniense*	Gut microbiota (Polynesia)	P1360	ND2	HG315672	2013 May	1	(*6*)
*Clostridium saudii*	Gut microbiota (Saudian obese)	P697	JCC	HG726039	2014 Aug	11	(*6**,**27*)
*Corynebacterium ihuae*	Gut microbiota (antimicrobials)	P892	GD6	JX424768	2013 May	3	(*6**,**28*)
*Corynebacterium lascolaense*	Urinary microbiota	P2174	MC3	LN881612	2013 Sep	6	(*6*)
*Corynebacterium phoceense*	Urinary microbiota	P1905	MC1	LN849777	2015 May	12	(*6**,**29*)
*Gabonia massiliensis*	Gut microbiota	P1910	GM3	LN849789	2017 Apr	1	(*6**,**30*)
*Nosocomicoccus massiliensis*	Gut microbiota (HIV)	P246	NP2	JX424771	2012 Feb	1	(*6**,**31*)
*Peptoniphilus grossensis*	Gut microbiota (obese)	P184	ph5	JN837491	2015 Nov	18	(*6**,**32*)
*Polynesia massiliensis*	Gut microbiota (Polynesia)	P1280	MS3	HF952920	2013 Mar	1	(*6*)
*Prevotella ihuae*	Gut microbiota (fresh feces)	P3385	Marseille-P3385T	LT631517	Not added	1	(*33*)
*Pseudomonas massiliensis*	Gut microbiota (Polynesia)	P1334	CB1	LK985396	2015 Apr	5	(*6**,**34*)
*Varibaculum timonense*	Gut microbiota (fresh feces)	P3369	Marseille-P3369T	LT797538	Not added	1	(*33*)

*Accession numbers indicate nucleotide sequences. CSUR, Collection de Souches de l’Unité des Rickettsies (an international strain collection).

Among the 105 colonies identified as new species isolated as a part of culturomics studies, identification was correct for 100 (95.2%) using MALDI-TOF mass spectrometry. Thus, 16S rDNA gene sequencing was required for 5 strains to achieve final identification. MALDI-TOF mass spectrometry was not able to provide a reliable identification for *Varibaculum timonense*, *Prevotella ihuae*, *Actinomyces ihuae*, and 2 *Corynebacterium phoceense* isolates. We confirmed identification of 9 supplementary strains, representing 5 species (*Corynebacterium lascolaense*, *Actinomyces ihuae*, *Corynebacterium ihuae*, *Nosocomiicoccus massiliensis*, and *Pseudomonas massiliensis*), using 16S rDNA gene sequencing (Tables 2,3). Overall, we sequenced 14 isolates, accounting for 8 species, for the 16S rDNA gene.

**Table 2:** Identification of bacterial pathogens by MALDI-TOF mass spectrometry, Marseille, France*

Species	MALDI-TOF identification (score)	Specimen	Duplicates per patient?†
*Actinomyces bouchesdurhonensis*	*Actinomyces bouchesdurhonensis* (1.85)	Pharynx swab	No
*A. bouchesdurhonensis*	*A. bouchesdurhonensis* (1.9)	Abscess	No
*Actinomyces ihuae*	*Actinomyces ihuae* (1.97)	Abscess	No
*A. ihuae*	*A. ihuae* (1.9)	Abscess	No
*A. ihuae*	*A. ihuae* (2.5)	Abscess	No
*A. ihuae*	*A. ihua*e (1.92)	Abscess	No
*A. ihuae*	*A. ihuae* (2.5)	Abscess	No
*A. ihuae*	*A. ihuae* (1.73)	Abscess	No
*A. ihuae*	*A. ihuae* (2.23)	Abscess	No
*A. ihuae*	*A. ihuae* (2.2)	Abscess	No
*A. ihuae*	*A. ihuae* (2.1)	Abscess	No
*A. ihuae*	*A. ihuae* (2.1)	Bone	No
*A. ihuae*	*A. ihuae* (2.52)	Puncture fluid	No
*A. ihuae*‡	*A. ihuae* (2.47)	Puncture fluid	No
*A. ihuae*‡	*Actinomyces* spp. (1.65)	Biopsy	No
*A. ihuae*‡	*A. ihuae* (2.32)	Abscess	No
*A. ihuae*‡	*A. ihuae* (2.33)	Abscess	No
*A. ihuae*‡	*A. ihuae* (1.95)	Puncture fluid	No
*A. ihuae*‡	*A. ihuae* (2.07)	Abscess	No
*Actinomyces marseillensis*	*Actinomyces marseillensis* (NA)	Blood culture	No
*Alistipes jeddahensis*	*Alistipes jeddahensis* (1.97)	Abscess	No
*Bacteroides timonensis*	*Bacteroides timonensi*s (1.95)	Blood culture	Yes
*B. timonensis*	*B. timonensis* (1.88)	Blood culture	Yes
*B. timonensis*	*B. timonensis* (1.96)	Blood culture	No
*Corynebacterium ihuae*‡	*Corynebacterium ihuae* (2)	Blood culture	No
*C. ihuae*	*C. ihuae* (2.2)	Wound	No
*C. ihuae*	*C. ihuae* (1.8)	Blood culture	No
*Corynebacterium lascolaense*	*Corynebacterium lascolaense* (2.2)	Urine	No
*C. lascolaense*	*C. lascolaense* (2.3)	Pacemaker	No
*C. lascolaense*	*C. lascolaense* (2.1)	Urine	Yes
*C. lascolaense*	*C. lascolaense* (2.14)	Urine	Yes
*C. lascolaense*‡	*C. lascolaensis* (2.2)	Urine	No
*Corynebacterium phoceense*	*Corynebacterium phoceense* (1.91)	Urine	No
*C. phoceense*	*Corynebacterium* spp. (2.3)	Unknown	No
*C. phoceense*	*C. phoceense* (2.6)	Blood culture	No
*C. phoceense*‡	No reliable identification	Blood culture	No
*Nosocomicoccus massiliensis*‡	*Nosocomicoccus massiliensis* (2.3)	Blood culture	No
*Peptinophilus grossensis*	*Peptinophilus grossensis* (2.1)	Abscess	No
*P. grossensis*	*P. grossensis* (2.18)	Biopsy	No
*P. grossensis*	*P. grossensis (*1.9)	Abscess	No
*P. grossensis*	*P. grossensis* (2.3)	Biopsy	No
*P. grossensis*	*P. grossensis* (1.9)	Biopsy	Yes
*P. grossensis*	*P. grossensis* (2.2)	Biopsy	Yes
*P. grossensis*	*P. grossensis* (2.18)	Biopsy	No
*P. grossensis*	*P. grossensis* (2)	Material	No
*P. grossensis*	*P. grossensis* (1.78)	Abscess	No
*P. grossensis*	*P. grossensis* (2.1)	Abscess	No
*P. grossensis*	*P. grossensis* (2.15)	Abscess	No
*P. grossensis*	*P. grossensis* (1.9)	Puncture fluid	No
*P. grossensis*	*P. grossensis* (2.1)	Puncture fluid	Yes
*P. grossensis*	*P. grossensi*s (2.3)	Puncture fluid	Yes
*P. grossensis*	*P. grossensis* (2.2)	Puncture fluid	No
*P. grossensis*	*P. grossensis* (2.1)	Abscess	Yes
*P. grossensis*	*P. grossensis* (2.1)	Abscess	Yes
*P. grossensis*	*P. grossensis* (1.9)	Puncture fluid	No
*P. grossensis*	*P. grossensis* (2.3)	Biopsy	No
*P. grossensis*	*P. grossensis* (2.31)	Biopsy	No
*P. grossensis*	*P. grossensis* (1.86)	Abscess	No
*Polynesia massiliensis*	*Polynesia massiliensis* (2.21)	Peritoneal fluid	No
*Prevotella ihuae*	No reliable identification	Abscess	No
*Pseudomonas massiliensis*	*Pseudomonas massiliensis* (2.5)	Blood culture	No
*Pseudomonas massiliensis*	*Pseudomonas massiliensis* (2)	Blood culture	No
*Pseudomonas massiliensis*‡	*Pseudomonas massiliensis* (1.9)	Blood culture	No
*Varibaculum timonense*	No reliable identification	Abscess	No
*MALDI-TOF, matrix-assisted laser desorption/ionization time-of-flight; NA, not available. †Replicated isolates in different specimens from the same patient. ‡Strains for which 16S rDNA sequencing was performed.			

**Table 3:** Identification of bacteria discovered as a part of culturomics studies in the clinical microbiology laboratory as commensals, Marseille, France*

Species	MALDI-TOF mass spectrometry identification (score)	Specimen	Duplicates per patient?†	Additional information
*Actinomyces bouchesdurhonensis*	*A. bouchesdurhonensis* (2)	Larynx biopsy	No	Polymicrobial
*Alistipes jeddahensis*	*Alistipes jeddahensis* (2.38)	Liquid feces	No	Seeking *Salmonella* spp.
*A. jeddahensis*	*A. jeddahensis* (2.45)	Liquid feces	No	Seeking *Salmonella* spp.
*A. jeddahensis*	*A. jeddahensis* (2.5)	Liquid feces	No	Seeking *Salmonella* spp.
*Anaerosalibacter massiliensis*	*Anaerosalibacter massiliensis* (1.78)	Rectal swab	No	Seeking MDR bacteria
*Butyricimonas phocaeensis*	*Butyricimonas phoaceensis* (2.36)	Liquid feces	No	Seeking toxigenic CD
*Clostridium culturomicsense*	*Clostridium culturomicsense* (2)	Liquid feces	No	Seeking toxigenic CD
*Clostridium jeddahtimonense*	*Clostridium jeddahtimonense* (2.1)	Liquid feces	No	Seeking toxigenic CD
*C. jeddahtimonense*	*C. jeddahtimonense* (2.3)	Liquid feces	No	Seeking toxigenic CD
*C. jeddahtimonense*	*C. jeddahtimonense* (2.4)	Liquid feces	No	Seeking toxigenic CD
*C. jeddahtimonense*	*C. jeddahtimonense* (2.1)	Liquid feces	No	Seeking toxigenic CD
*C. jeddahtimonense*	*C. jeddahtimonense* (2.2)	Liquid feces	Yes	Seeking toxigenic CD
*C. jeddahtimonense*	*C. jeddahtimonense* (1.72)	Liquid feces	Yes	Seeking toxigenic CD
*C. jeddahtimonense*	*C. jeddahtimonense* (2.3)	Liquid feces	No	Seeking toxigenic CD
*C. jeddahtimonense*	*C. jeddahtimonense* (2.1)	Liquid feces	No	Seeking toxigenic CD
*Clostridium massilioamazoniense*	*Clostridium massilioamazoniense* (1.7)	Liquid feces	No	Seeking toxigenic CD
*Clostridium saudii*	*Clostridium saudii* (2.5)	Liquid feces	No	Seeking toxigenic CD
*C. saudii*	*C. saudii* (1.74)	Liquid feces	No	Seeking toxigenic CD
*C. saudii*	*C. saudii* (2.5)	Liquid feces	No	Seeking toxigenic CD
*C. saudii*	*C. saudii* (1.94)	Liquid feces	No	Seeking toxigenic CD
*C. saudii*	*C. saudii* (2.18)	Liquid feces	No	Seeking toxigenic CD
*C. saudii*	*C. saudii* (1.77)	Liquid feces	No	Seeking toxigenic CD
*C. saudii*	*C. saudii* (1.93)	Liquid feces	No	Seeking toxigenic CD
*C. saudii*	*C. saudii* (2.1)	Liquid feces	No	Seeking toxigenic CD
*C. saudii*	*C. saudii* (1.9)	Liquid feces	No	Seeking toxigenic CD
*C. saudii*	*C. saudii* (2.47)	Liquid feces	No	Seeking toxigenic CD
*C. saudii*	*C. saudii* (1.83)	Liquid feces	No	Seeking toxigenic CD
*Corynebacterium lascolaense*	*Corynebacterium lascolaense* (1.85)	Intrauterine device	No	Not considered
*C. lascolaense*	*C. lascolaense* (2.1)	Urine	No	Growth not significant
*Corynebacterium phoceense*	*Corynebacterium phoceense* (2.1)	Vagina	No	Not considered
*C. phoceense*	*C. phoceense* (1.9)	Vagina	No	Not considered
*C. phoceense*	*C. phoceense* (2.1)	Vagina	No	Not considered
*C. phoceense*	*C. phoceense* (2)	Vagina	No	Not considered
*C. phoceense*	*C. phoceens*e (2.2)	Vagina	No	Not considered
*C. phoceense*	*C. phoceense* (2)	Vagina	No	Polymicrobial
*C. phoceense*	*C. phoceense* (2.2)	Vagina	No	Not considered
*C. phoceense*	*C. phoceense* (1.97)	Urine	No	Polymicrobial
*Gabonia massiliensis*	*Gabonia massiliensis* (2.3)	Liquid feces	No	Seeking MDR bacteria
*Pseudomonas massiliensis*	*Pseudomonas massiliensis* (2)	Skin swab	Yes	Seeking *S. aureus* carriage
*P. massiliensis*	*P. massiliensis* (2.5)	Skin swab	Yes	Seeking *S. aureus* carriage
*MALDI-TOF, matrix-assisted laser desorption/ionization time-of-flight. †Replicated isolates in different specimens from the same patient.				

### Species Potentially Relevant as Human Pathogens

Among the 105 isolates included in this work, 64 were isolated as potential pathogens, accounting for 14 different species. Most were anaerobes that were cultured from abscesses or punctures, often involved in cases of polymicrobial infections. *Peptoniphilus grossensis* (18 cases) and *Actinomyces ihuae* (17 cases) were the most commonly isolated bacteria (Table 1). These species were initially cultured from the human gut. Special attention was given to *A. ihuae* infections (Table 4), which were strongly associated with breast abscess or genital area infections. Also, 10 bacteremia-involved species were isolated as a part of culturomics studies. *Bacteroides timonensis* was thus isolated in 3 blood cultures from 2 patients, whereas *Pseudomonas massiliensis* was found in 3 bacteremia episodes. *Corynebacterium phoceense* and *Corynebacterium ihuae* were recovered from 2 bloodstream infection episodes, whereas *Actinomyces marseillensis* and *Nosocomicoccus massiliensis* were each isolated from 1 blood sample (from 2 different patients). *B. timonensis*, *P. massiliensis*, *N. massiliensis*, and *C. ihuae* were first cultured from the human gut, whereas *A. marseillensis* was first isolated from respiratory microbiota and *C. phoceense* was first isolated from urinary microbiota. Overall, species cultured as part of culturomics studies were found to be potential pathogens in 59 different patients (Table 2). The significance of the presence of *P. massiliensis* in a lens from a patient with keratitis was ultimately not interpreted.

**Table 4:** Characteristics of 17 persons with *A. ihuae* infection, Marseille, France, April 2015–March 2018*

Patient no.	Patient age, y/sex	Sampling site	Incubation time, h	Culture result	MALDI-TOF mass spectrometry score	16S rRNA result
1	24/F	Periareolar right breast	48	Polymicrobial	2.54	NA
2	26/F	Umbilical collection	48	Pure	2.5	NA
3	37/M	Periareolar left breast	72	Polymicrobial	1.97	NA
4	33/F	Breast	72	Polymicrobial	2.1	NA
5	77/F	Bone	72	Polymicrobial	2.1	NA
6	22/M	Testicular collection	96	Pure	1.95	*A. ihuae* 99.70%
7	56/M	Back	48	Pure	2.32	*A. ihuae* 99.70%
8	55/F	Labia majora	72	Polymicrobial	2.07	*A. ihuae* 99.70%
9	30/F	Labia majora	72	Pure	2.47	*A. ihuae* 99.70%
10	26/F	Labia majora	72	Polymicrobial	2.33	*A. ihuae* 99.60%
11	44/M	Leg ulcer	48	Polymicrobial	NA	*A. ihuae* 99.50%
12	66/M	Cervical collection	72	Polymicrobial	2.23	NA
13	49/M	Superinfected sebaceous cyst	48	Polymicrobial	1.9	NA
14	18/F	Sacrococcygeal cyst	96	Pure	2.45	NA
15	26/F	Labia majora	72	Polymicrobial	2.2	NA
16	45/F	Breast abcess	72	Polymicrobial	1.73	NA
17	44/M	Axillar abcess	96	Polymicrobial	1.92	NA
* MALDI-TOF, matrix-assisted laser desorption/ionization time-of-flight; NA, not available						

### Species Isolated as Human Commensal Members

In this work, 40 isolates corresponding to 12 species discovered as a part of culturomics studies were isolated as belonging to the human flora. Of these, 22 were recovered when evaluating for toxigenic *C. difficile*, following a positive result with the GeneXpert *C. difficile* test. *C. saudii* was isolated in this context 11 times, followed by *C. jeddahtimonense* (8 times), *C. culturomicsense*, *Butyricimonas phocaeensis*, and *Anaerosalibacter massiliensis* (1 time each) (Table 3). These 5 species were first cultured from fecal specimens (Table 1).

In addition, *Corynebacterium lascolaense* was identified in 1 urine specimen, but in an insufficient quantity to be considered clinically relevant. Similarly, *C. phoceense* was recovered from 1 urine sample and from 7 vaginal swabs but was never reported to a physician in this context. These species were first cultured from urinary microbiota. Finally, *Pseudomonas massiliensis*, which was cultured from the human gut, was also recovered twice from skin swabs collected from the same physician after an epidemiologic investigation. Overall, species cultured for the first time as a part of culturomics studies were found as commensals in 38 different patients.

## Discussion

This work constitutes the proof of concept that exploration of the repertoire of commensal bacteria enables identification of microorganisms involved in clinical microbiology. Indeed, the strategy of combining high-throughput culture techniques, MALDI-TOF mass spectrometry identification, and 16S rDNA gene sequencing of misidentified isolates enabled us to add 306 spectral references for 292 different new bacterial species to our laboratory’s database. Thus, with culturomics, 21 new species were identified 105 times, in 98 patients. The results are robust; identification scores were all >1.9 with exclusion of multiple identifications. In addition, identification of 9 strains using 16S rDNA sequencing, accounting for 5 species, confirmed the initial recognition by MALDI-TOF mass spectrometry (Table 2). These results strengthen our belief that identifying commensal microbes provides a valuable contribution to clinical microbiology, as revealed by the decrease in the number of unidentified colonies by MALDI-TOF mass spectrometry over time (Figure).

As exemplified for *A. ihuae* infections (Table 4), these microorganisms, which were isolated mainly from the human gut, can probably be found frequently in polymicrobial cultures. Thus, the microbiologist may be tempted to abandon the final identification of a microorganism found in such a situation, concluding that the infection is polymicrobial.

The extension of the bacterial repertoire associated with humans will considerably increase the number of bacteria associated with human diseases. In this study alone, over a 5-year period, 2.8% (21/744) of the overall identified bacteria would not have been identified without incrementing the MALDI-TOF mass spectrometry database with spectra obtained from culturomics studies. 

On the whole, pathogenic microbes are also often found as commensals, as is currently well known for *C. difficile*, *S. aureus*, and *S. pneumoniae* (*35**–**37*)*.* In our study, for example, *Corynebacterium phoceense*, *Pseudomonas massiliensis*, and *C. lascolaense* were found as both commensals and pathogens. This finding highlights the need for establishment of a repertoire of human microbes (*38*), which was recently estimated at 2,776 species, of which more than 10% were recovered by culturomics studies. Such a repertoire of prokaryotes associated with humans not only benefits microbiota studies, through notation of unknown sequences with new species genome sequencing, but also enables studying the role of these species in human infections (*39*)*.* We estimate that, among the cases included here, the presence of species cultured as part of culturomics studies was potentially clinically relevant for 60 of them (61.2%). The online availability of the MALDI-TOF mass spectrometry spectra obtained from these species discovered by culturomics (http://www.mediterranee-infection.com/article.php?laref=256&titre=urms-database) ensures their further identification by other laboratories.

Culturomics was initially designed to exhaustively identify commensals inhabiting human surfaces and thus can potentially lead in the future to personal medical interventions as a part of microbiome studies. However, the thinnest barrier between commensalism and pathogenicity, which should lead researchers to rethink Koch’s postulate (*40*)*,* has rendered culturomics studies useful in the field of clinical microbiology despite a potential skepticism. We show herein that, while contributing to the extension of the bacterial repertoire associated with humans, culturomics studies also enlarge the spectrum of prokaryotes involved in infectious diseases.
